# Cause-specific mortality after spousal bereavement in a Danish register-based cohort

**DOI:** 10.1038/s41598-025-90657-1

**Published:** 2025-02-20

**Authors:** Mathilde Marie Brünnich Sloth, Johannes Hruza, Laust Hvas Mortensen, Samir Bhatt, Alexandros Katsiferis

**Affiliations:** 1 Section of Epidemiology, Department of Public Health, University of Copenhagen, Copenhagen, Denmark; 2 Data Science Lab, Statistics Denmark, Copenhagen, Denmark; 3 Department of Infectious Disease Epidemiology, Imperial College London, London, UK; 4 The ROCKWOOL Foundation, Ny Kongensgade 6, 1472, Copenhagen K, Copenhagen, Denmark

**Keywords:** Spousal bereavement, Cause-specific mortality, Cohort study, Counterfactuals, Machine learning, Prediction, Diseases, Health care, Risk factors

## Abstract

Spousal bereavement is associated with increased all-cause mortality, but less is known for cause-specific mortality. We estimated the average effect of bereavement on cause-specific mortality. Furthermore, we developed and evaluated the performance of prediction models estimating individualized mortality risks. This matched cohort study leveraged nationwide registry data on 223,500 married Danish individuals aged ≥ 65 years. Individuals were followed from bereavement date until death, emigration, or 3-year end of follow-up. G-estimation was used to estimate the average effect of bereavement on cause-specific mortality. Risk prediction models were developed and internally validated using logistic regression and Extreme Gradient Boosting utilizing information on sociodemographic factors and healthcare expenditures prior to bereavement. Among males, bereavement was associated with increased 3-year mortality from cardiovascular disease (CVD) (Risk Difference: 8 deaths/1000 individuals [95% confidence intervals (CI) 3;13]), digestive diseases (3 [95% CI 1;5]), psychiatric diseases/suicide (3 [95% CI 1;5]), and respiratory diseases (4 [95% CI 1;8]) and decreased 3-year mortality for dying from dementia/Parkinson’s disease (-4 [95% CI -6;-2]). Among females, bereavement was only associated with increased 3-year mortality from CVD (5 [95% CI 3;7 ]) and from psychiatric diseases/suicide (1 [95% CI 1;2]). The prediction models developed using sociodemographic information and healthcare expenditures showed limited accuracy in identifying cause-specific mortality risk. On average, spousal bereavement slightly elevated the risk for most causes of death for males and less so for females. Despite incorporating comprehensive healthcare and sociodemographic data, accurately predicting individual cause-specific mortality remains challenging.

**Supplementary Information:**

The online version contains supplementary material available at 10.1038/s41598-025-90657-1.

## Introduction

Spousal bereavement i.e. the loss of a spouse is a stressor often occurring in old age^1^. As individuals age, they become frailer and more susceptible to stressors having an impact on their health and longevity^2^. With an aging society, spousal bereavement becomes increasingly important to investigate. Spousal bereavement has been found to have far-reaching consequences for health. This event has been associated with various unfavorable health outcomes, such as low diet quality^3^, depression^4^, and mortality^5^. This underscores the urgency of understanding and addressing the impact of this life-altering experience.

Spousal bereavement has consistently been found to increase the mortality risk among spousal bereaved individuals in previous studies^6–16^, generally with males showing a higher mortality following bereavement compared to females. Only a few studies have investigated cause-specific mortality in older adults^13,15–17^. These studies reported higher hazard rates for spousal bereaved older adults of dying from malignant cancer, respiratory diseases, circulatory diseases, and external causes compared to non-bereaved older adults^13,15^. However, these studies have investigated the link between spousal bereavement and cause-specific mortality with regression analysis estimating the magnitude of the association without using a causal framework. Furthermore, there is a lack of studies investigating the magnitude of the association on an absolute scale.

Recently, researchers have taken this knowledge a step further by developing prognostic prediction models that predicted all-cause mortality after spousal bereavement^10,18^. An advantage of prognostic prediction models is that this method facilitates individual prediction of cause-specific risks whereas most previous research on spousal bereavement^6–9,11–16^ has used methods that estimates the average association between bereavement and all-cause mortality. Prediction models that are validated thoroughly have practical applications and can be used for resource prioritization by providing risk scores that help target those at high risk. Previous prognostic prediction models initiated at time of bereavement has found information on healthcare expenditures being particularly predictive of all-cause mortality^10,18^. No study has to our knowledge investigated whether healthcare expenditures prior to spousal bereavement are predictive of cause-specific mortality. The current study aims to fill this gap in knowledge, building upon the aforementioned studies^10,18^.

In a large cohort of Danish spousal bereaved older adults matched with their non-bereaved married counterparts we first aim to investigate the association between spousal bereavement and cause-specific mortality. We employed a potential outcome model^19^ to a causal inference analysis to ascertain whether there was a sex-specific association on average between spousal bereavement and cause-specific mortality. Secondly, we developed and internally validated prognostic models for cause-specific mortality within three years after bereavement. We used the Danish registers to include information on socioeconomic factors and health determinants including different types of healthcare expenditures as possible predictors in the models. In sum, this study aims to advance our understanding of spousal bereavement as a major life stressor and cause-specific mortality.

## Methods

### Study design

**Fig. 1:**
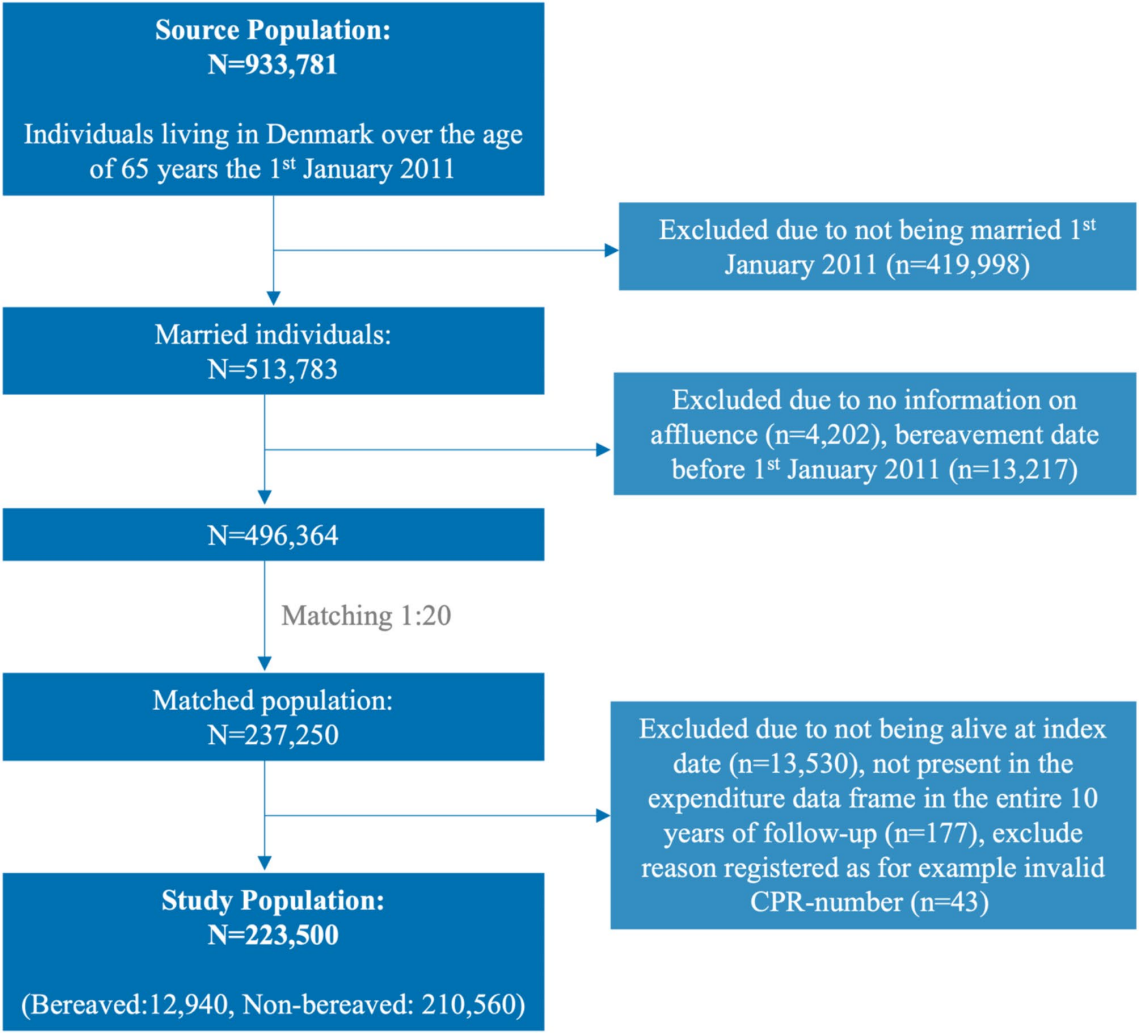
Flow chart of the study population.

The original population consisted of all individuals aged 65 years and above residing in Denmark as of January 1st, 2011. Information on the setting is presented in Supplementary Text 1. We used a matched cohort study design by matching each individual experiencing spousal bereavement in 2012 with on average 20 non-bereaved controls based on a set of sociodemographic and overall health-status information at the date of bereavement (index date). Both groups were followed up from the index date until death, emigration or up to a maximum period of 3-years (administrative censoring), whichever comes first. The final matched cohort consisted of 223,500 married individuals, of which 12,940 (5.8%) experienced bereavement in 2012. The selection process and eligibility criteria of the study are described in Fig. 1.

### Spousal bereavement

Spousal bereavement was defined as the loss of one’s spouse. The bereavement status was included as a binary variable to determine if individuals had experienced bereavement in the year of 2012. The date of bereavement was used as time zero in the prediction analysis, i.e., the time where the prediction for an individual is being given. Information on bereavement was retrieved form the Danish Civil Registration System.

### Cause-specific mortality

The outcome of the analysis was cause-specific mortality. Information on death were retrieved from the Danish Register of Causes of Death^20^. In this study we used the Automated Classification of Medical Entities (ACME) to determine the cause of death, which is based on the causes reported on the death certificate. ACME applies the World Health Organization (WHO) rules to the International Classification of Diseases (ICD) codes and selects the underlying cause of death^21^. The following causes of death were included: dying from (1) cancer, (2) CVD, (3) diabetes, (4) dementia or Parkinson disease, (5) digestive diseases, (6) psychiatric diseases or suicide, (7) respiratory diseases and (8) other causes of death. The ICD-10 codes for each cause of death are presented in Supplementary Table 1.

### Confounders and predictors

Age, sex, immigration status, affluence index, number of children and number of comorbidities measured at baseline (January 1,2011), were included as potential confounders and predictors. This information was sourced from the Danish Civil Registration System and the National Patient Register. We additionally used longitudinal data on the healthcare expenditures of individuals 1-year prior to the index date (date of bereavement or for matched non-bereaved the date of bereavement of the matched bereaved) as another source of information potentially predictive of mortality. The Directed Acyclic Graph describing our analysis is illustrated in Supplementary Fig. 2.

Age was categorized into five-year intervals: 65–69, 70–74, 75–79, 80–84, or ≥ 85 years. Immigration status was dichotomized into 1: Danish citizens or 2: immigrants and descendants. The number of children was categorized as follows: none, 1, 2, 3, or ≥ 4 children. Comorbidities were categorized into the following groups: none, 1, 2, 3, or ≥ 4 comorbidities based on data from the creation of the National Patient Register in 1994 until the 1st January 2011.The affluence index, utilized as a measure of socioeconomic status, was derived from an individual’s wealth and income based on a previous paper by Hansen and colleagues^22^. The healthcare expenditures entailed longitudinal information on prescription, hospital (in- and outpatient), primary care, residential care, and home care expenses. The healthcare expenditures used for this study is based on the data used in the paper by Hansen and colleagues^22^ and made available from Statistics Denmark. The Danish healthcare system is primarily funded by taxes, which means that the information on medical spending retrieved from the registers accounts for 97% of the spending^23^. Each type of healthcare expenditure was based on weekly data in 1000s of Danish kroner (DKK) calculated as a weekly average the year prior to index date. The expenditures were categorized into four categories for the inferential analysis (the average effect of bereavement). The lowest category was total weekly average of healthcare (1) Less than 10 DKK/week and (2) 10–99 DKK/week, (3) 100–499 DKK/week, and lastly (4) the highest spending group of 500 DKK/week or more (10 DKK = 1,33 €). For the prediction modelling workflow, the healthcare expenditures were included separately as continuous predictors.

### Matching

Matching was used to create comparable groups of bereaved and non-bereaved individuals with shared follow-up periods. Logistic regression propensity score matching was utilized to match each bereaved individual with approximately 20 non-bereaved controls at the date of bereavement (index date) without replacement. The matching criteria included age, sex, number of children, number of comorbidities, immigration status, and affluence index as of January 1, 2011. We excluded controls who did not survive up to the index date, for comparability purposes. Non bereaved individuals were allowed to experience the stressor after the index date. The balance of matching was assessed by evaluating standardized mean differences (SMD), with a threshold of < 0.1 indicating balance among covariates. The balance of the matching is presented in Supplementary Fig. 1 (a, b). Mahalanobis distance matching was additionally tested but did not reduce the SMD of the covariates among the two groups.

### Statistical methods

#### The average effect of bereavement

The analysis aimed to estimate the average effect of bereavement on dying from each specific cause for older adults. We performed the analysis using an average treatment effect (ATE) model with a cause specific cox model to account for the competing risk of dying from another cause^24^. Spousal bereavement was considered the treatment in the model (spousal bereavement A = 1, non-bereavement A = 0). The average *bereavement* effect was estimated via the *ate* function in R from the *RiskRegression* package, using a G-estimation approach, grounded in the assessment of counterfactual scenarios (also referred to as a potential outcome model). The ATE for the right censored event time T is given by

$$ATE=\mathbb{E}\left[ {\mathbb{E}\left[ {Y|{{A}}=1,{{X}}} \right] - {\mathbb{E}}\left[ {{{Y|A}}=0,{{X}}} \right]} \right]{\text{}}$$

In this study, we let the covariates X be distributed by the sample distribution of the matched population. The estimator for the ATE uses two cox models for competing risks of death (The first model (j = 1) predicts the death of interest, and the second model (j = 2) predicts death of other causes). The two cox models are given by

$$\:{\lambda\:}_{j}\left(t\:\right|\:\:\text{A},\:\text{X})\:=\:\:{\lambda\:}_{j0}(t)\:{e}^{{\beta\:}_{j}X+{\gamma\:}_{j}A}$$

The quantity $\mathbb{E}\left[ {Y|{\text{A}}={\text{i}},{\text{X}}} \right]$ for $\:i\:\in\:\left\{\text{0,1}\right\}$ is estimated by a cox model for competing risk:

$$\hat {\mathbb{E}}\left[ {Y|{\text{A}}={\text{i}},{\text{X}}} \right]=\mathop \smallint \limits_{0}^{T} \hat {\lambda }\left( {s|A=i,X} \right)\hat {S}\left( {s - |A=i,X} \right)ds$$

where $\hat {\lambda }\left( {s|A=i,X} \right)={\hat {\lambda }_{01}}\left( s \right){e^{\widehat {{{\beta _1}}}X~+~i{{\hat {\gamma }}_1}}}$ is the cause-specific hazard function of the event happening at time s and $\hat {S}\left( {s~ - |A=i,X} \right)={e^{ - \mathop \smallint \limits_{0}^{{s - }} {{\hat {\lambda }}_{10}}\left( t \right){e^{\widehat {{{\beta _1}}}X~+~i\widehat {{{\gamma _1}}}}}+\widehat {{{\lambda _{20}}}}\left( t \right){e^{\widehat {{{\beta _2}}}X~+~i\widehat {{{\gamma _2}}}}}dt}}$ is the event-free survival function.

In this case the average bereavement effect estimates the difference in the average outcome in the case where the whole population is non-bereaved in contrast to the whole population being bereaved. The average bereavement effect is presented in absolute risk differences per 1,000 individuals with corresponding 95% confidence intervals. The confidence intervals were computed using 1,000 bootstraps.

The older adults could either experience 0: not dying (alive), 1: dying from a specific cause, or 2: dying from another cause. The analysis was performed for males and females separately. All analysis were adjusted for the confounders: age, immigration status, affluence index, number of children, number of comorbidities, and healthcare expenditures. In the modelling, we assumed no unmeasured confounding.

#### Prediction

We restricted the cohort to older adults who had experienced bereavement (*N* = 12,940) for the prediction analysis. We developed prediction models and assessed the prognostic potential of various predictors. We trained the models on 75% of the data and tested on the remaining unseen 25%. For each cause of death, five different prediction models were developed. The first four models were based on a logistic regression model (LRM) with increased number of predictors for each model. The first model was a null model, which assigned the same risk for all individuals in the population based on the mean prevalence of the outcome in the training set (from here on referred to as LRM null). The second model was the simple model with the predictors: age at bereavement, and sex (from here on referred to as LRM simple). The third model included the predictors: age at bereavement, sex affluence index, number of children, number of comorbidities (from here on referred to as LRM socio + comorbidity). The fourth model included the predictors: age at bereavement, sex, immigration status, affluence index, number of children, number of comorbidities, and each type of healthcare expenditures (prescription, primary care, inpatient and outpatient hospital care, home care, residential care) (from here on referred to as LRM full). This model included restricted cubic splines with three knots to allow for a non-linear relationship between healthcare expenditures and the risk of dying from a specific cause.

We further assessed whether a more flexible machine learning model could increase the predictive capacity of our task compared to the logistic regression model. Specifically, we developed such models using the eXtreme Gradient Boosting (XGBoost) algorithm, which allowed for non-linear effects and interactions between all available predictors. The XGBoost model is a tree-based method that uses decision trees and gradient boosting to create predictions. The XGBoost model included, age at bereavement, sex, immigration status, affluence index, number of children, number of comorbidities, and each healthcare expenditures as predictors (from here on referred to as XGBoost full). For the tuning of hyperparameters for XGBoost we used a 5-fold cross-validation split of the training data. Race ANOVA^25^ was used as the tuning method to efficiently tune the number of trees, tree depth, number of predictions randomly sampled at each split, minimum number of data points in a node that can be split further, number of iterations that will be performed without improvement and then the learning rate. The combination of hyperparameters that gave the lowest Brier Score was then used for the XGBoost model. We used a maximum entropy design, allowing 30 different candidate values to be tested for each hyperparameter.

The validation of the prediction models was evaluated with the area under the receiver operating characteristic curve (AUC), the Brier Score, the IPA (Index of Predictive Accuracy), calibration plots and potential clinical benefit via decision curve analysis. The AUC quantifies the model’s discriminatory ability, measuring the probability of assigning higher risks to individuals with the target outcome compared to those without^26^.

The Brier Score provides an estimate of the overall prediction error by incorporating both discrimination and calibration. It is defined as the mean squared difference between the predicted probabilities and the actual outcomes. A lower Brier Score indicates superior predictive performance, with a score of 0 representing perfect prediction accuracy, and a score of 1 reflecting complete inaccuracy. The formula for calculating the Brier Score is as follows^27^:

$$\:\text{Brier Score}=\frac{1}{N} \sum\:_{i=1}^{N}{\left(\text{prediction}_{i}-\text{outcome}_{i}\right)}^{2}$$

The Index of Prediction Accuracy (IPA) measures the overall predictive performance of a model relative to a null model. The null model disregards all predictor variables and assigns the same probability of the outcome to all individuals, corresponding to the prevalence of the outcome in the population. The formula for the IPA is as follows^28^:

$$\:\text{IPA}=1-\frac{\text{Brier Score(Prediction Model})}{\text{Brier Score(Null Model})}$$

The decision curve analysis evaluates the potential clinical utility of a prediction model by quantifying its net benefit across a range of threshold probabilities. This method assesses whether the model improves decision-making compared to default strategies, such as treating all individuals or none, based on varying levels of predicted risk. It integrates the trade-offs between the benefits of true-positive predictions and the harms of false positives - the latter weighted by the odds of the specific threshold probability. More specifically, the formula for the Net Benefit is as follows^29^:

$$\text{Net Benefit}=\frac{\text{True Positives}}{N}-\frac{\text{False Negatives}}{N}\times\:\frac{{p}_{t}}{1-{p}_{t}}$$

The comparison of the five prediction models based on the mentioned metrics were performed as a stepwise comparison between a more complex model and a simpler one. A delta AUC or Brier Score confidence intervals that did not overlap 0 was considered to reflect a difference in the two rival models being compared. The metrics are presented in percentages (estimates are multiplied by 100). The decision curve analysis was performed to investigate the net benefit at different risk thresholds for each model to gain more insights on clinical utility.

#### Software

The code is available at GitHub.

## Results

### Descriptive analysis

Table 1 shows the distribution of baseline characteristics. The study population consisted of a higher proportion of females (61.6%) than males. The age distribution within the study population showed that 20.0% of individuals fell within the 65–69 years range, 27.9% within the 70–74 years range, 24.7% within the 75–79 years range, 16.5% within the 80–84 years range, and 10.9% were aged 85 years or older. Furthermore, the study population was primarily comprised of individuals with Danish origin, accounting for 96.5% of the cohort, while immigrants and their descendants made up the remaining 3.5%. The majority (63.4%) had one or more diseases, with 36.6% without a hospital diagnosis for a disease. The older adults mostly had children, with two children being the most common. The vast majority (76.1%) of the older adults had an average healthcare expenditure of 10–99 DKK/week the year prior to the index date.

**Table 1:** Characteristics of the study population in *n(%)* (*N* = 223,500).

Variable		Total (*n* = 223,500)
Bereavement status	Non-bereaved	210,560 (94.2)
	Bereaved	12,940 (5.8)
Sex	Males	85,882 (38.4)
	Females	137,618 (61.6)
Age at bereavement	65–69 years	44,683 (20.0)
	70–74 years	62,253 (27.9)
	75–79 years	55,244 (24.7)
	80–84 years	36,949 (16.5)
	≥ 85 years	24,371 (10.9)
Immigration status	Danish	215,596 (96.5)
	Immigrants or descendants	7,904 (3.5)
Number of comorbidities	No comorbidities	81,853 (36.6)
	1	56,907 (25.5)
	2	36,287 (16.2)
	3	22,018 (9.9)
	≥ 4 comorbidities	26,435 (11.8)
Number of children	No children	25,598 (11.5)
	1	47,675 (21.3)
	2	87,113 (39.0)
	3	44,964 (20.1)
	≥ 4 children	18,150 (8.1)
Affluence index	Lowest	63,531 (28.4)
	Second	55,591 (24.9)
	Third	56,883 (25.5)
	Highest	47,495 (21.3)
Average healthcare expenditures	Less than 10 DKK/week	17,910 (8.0)
	10–99 DKK/week	170,181 (76.1)
	100–499 DKK/week	26,751 (12.0)
	More than 500 DKK/week	8,658 (3.9)

### The average bereavement effect

Table 2 shows the absolute risks and risk differences for the average bereavement effect on cause-specific mortality within three years for older males and females.

Most of the older adults remained alive during the 3-year follow-up. In total 27,548 older adults died within 3 years of the index date equivalent to 12.3%, with the most common cause of death being cancer and the lowest being psychiatric diseases or suicide. Supplementary Table 2 provides information on the most common causes of death within in cause.

Among older males, the absolute risks of dying from cardiovascular diseases for both older adults who had experienced spousal bereavement (60 deaths/1000 individuals) and older adults who had not experienced bereavement (52 deaths/1000 individuals) showed a risk difference of 8 [95% CI 3;13] additional deaths associated with spousal bereavement per 1000 individuals. For dying from digestive diseases and psychiatric diseases or suicide the results showed smaller absolute risks for both older males who had and had not experienced bereavement with a risk difference of 3 [95% CI 1;5] additional death associated with experiencing bereavement per 1000 individuals. The absolute risk of dying from respiratory diseases were 24 and 28 deaths per 1000 individuals for older males who had not and had experienced bereavement, respectively. Experiencing spousal bereavement was associated with 4 [95% CI 1;8] additional deaths due to respiratory diseases per 1000 individuals compared to older adults who had not experienced bereavement. Experiencing spousal bereavement showed different findings for dying from dementia or Parkinson’s, where bereavement was associated with a negative risk difference of 4 less deaths per 1000 individuals [95% CI -6;-2] compared to not experiencing spousal bereavement.

The results for older females showed lower absolute risks. The absolute risks for dying from cardiovascular diseases were less than half of males, with 20 deaths due to cardiovascular diseases for older females who had not experienced bereavement and 25 deaths for older females who had experienced bereavement. Among older females, experiencing bereavement was associated with an additional 5 [95% CI 3;7] deaths due to CVD per 1000 individuals. The absolute risks for dying from psychiatric disease or suicide was 1 death of this cause per 1000 older adults who had not experienced bereavement and 2 deaths for older adults who had experienced bereavement with a risk difference of 1 [95% CI 1;2] deaths associated with bereavement per 1000 individuals.

The results indicated differences in the risk of dying from cancer, dementia or Parkinson’s disease, and diabetes for older adults who had compared to had not experienced bereavement for both males and females. For older females, we additionally found no risk difference for dying from digestive diseases and respiratory diseases.

**Table 2:** The average bereavement effect on cause-specific death among older adults presented as absolute risks for bereaved and non-bereaved older adults in deaths per 1,000 individuals.

Cause of death	Absolute risks [95% CI]		Risk difference [95%CI]
	Non-bereaved	Bereaved	
Males (*N* = 85,882)			
Cancer (Observed number of deaths = 4,523)	53 [51;54]	54 [49;60]	1 [− 4;7]
Cardiovascular disease (Observed number of deaths = 4,546)	52 [51;54]	60 [55;65]	**8 [3;13]**
Dementia or Parkinson’s disease (observed number of deaths = 1,366)	16 [15;17]	12 [10;15]	− **4 [**− **6;** − **2]**
Diabetes (Observed number of deaths = 374)	4 [4;5]	5 [4;7]	1 [0;3]
Digestive diseases (Observed number of deaths = 447)	5 [5;6]	8 [6;11]	**3 [1;5]**
Psychiatric diseases or suicide (Observed number of deaths = 121)	1 [1;1]	4 [3;6]	**3 [1;5]**
Respiratory diseases (Observed number of deaths = 2,093)	24 [23;25]	28 [25;32]	**4 [1;8]**
Females (*N* = 137,618)			
Cancer (Observed number of deaths = 3,972)	29 [28;30]	27 [24;29]	− 3 [− 5;0]
Cardiovascular disease (Observed number of deaths = 2,825)	20 [19;21]	25 [23;27]	**5 [3;7]**
Dementia or Parkinson’s disease (Observed number of deaths = 1,197)	9 [8;9]	8 [7;9]	− 1 [− 2;0]
Diabetes (Observed number of deaths = 235)	2 [1;2]	2 [1;3]	0 [0;1]
Digestive diseases (Observed number of deaths = 438)	3 [3;3]	4 [3;5]	1 [0;2]
Psychiatric diseases or suicide (Observed number of deaths = 94)	1 [0;1]	2 [1;3]	**1 [1;2]**
Respiratory diseases (Observed number of deaths = 1,484)	11 [10;11]	12 [10;14]	1 [0;3]

Bold risk difference estimates indicate estimates with confidence intervals not overlapping zero.

All models were adjusted age, immigration status, affluence index, number of children, number of comorbidities and healthcare expenditures.

### Predicting cause-specific mortality

We assessed the predictive value of sociodemographic variables and healthcare expenditures for cause-specific mortality within 3 years of spousal bereavement in the restricted data of 12,940 bereaved individuals. Supplementary Fig. 3 shows the AUC, the Brier Scores, and IPAs for all models. The predictive performance varied substantially across different causes of death. The full models showed the highest discriminative ability for dying from diabetes (AUC 88.62% [95% CI 83.31;93.92%]), dementia or Parkinson’s disease (AUC 87.08% [95% CI 82.35; 91.80%]), and respiratory diseases (AUC 81.86% [95% CI 78.03; 85.68%]).

**Fig. 2:**
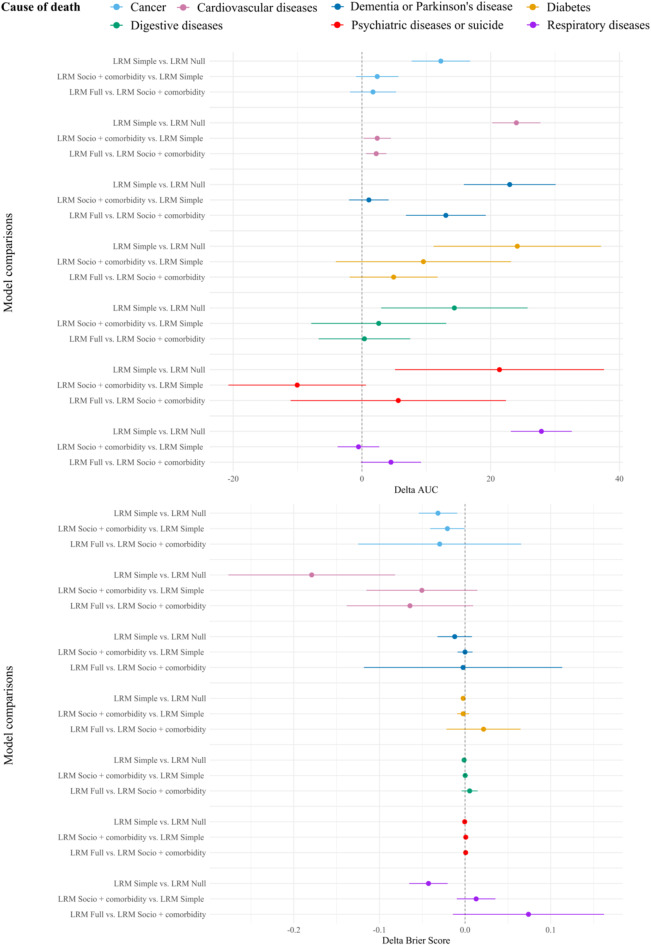
Model comparison evaluated with delta area under the receiver (AUC) and delta Brier Score with corresponding 95% confidence intervals.

Model comparisons are shown in Fig. 2 which illustrates the differences in the AUC and Brier Score (see Supplementary Table 4 for estimates and 95% CI). The delta AUC shows improved discrimination in all causes of death when comparing the LRM simple to the LRM Null model. Furthermore, adding information on sociodemograhic variables and comorbidity did not improve the discrimination apart from dying from CVD (ΔAUC: 2.38 [95% CI 0.26; 4.50]. For dying from CVD adding healthcare expenditures as a predictor also further improved discrimination with a difference in AUC of 2.22 [95% CI 0.65; 3.80] when comparing LRM Full to LRM socio + comorbidities. However, when investigating the prediction accuracy the delta Brier scores showed no indication that adding information on sociodemographic variables and healthcare expenditure improved the predictive performance for all causes of death. For example, when comparing the LRM Full to the LRM socio + comorbidities for dying from cancer the delta Brier score was − 0.03 [95% CI − 0.12; 0.07] indicating no difference in performance.

### Machine learning models versus classic models

We compared the XGBoost models against our logistic regression models with all predictors. Across all causes of death, the differences in prediction accuracy were not significant, with all Delta Brier Score confidence intervals including zero. The differences ranged from − 0.07 (95% CI: -0.15 to 0.00) for dying from respiratory diseases to 0.05 (95% CI: -0.02 to 0.11) for dying from CVD, indicating that the more complex machine learning approach did not improve upon the logistic regression models with the same predictors. Supplementary Fig. 3 shows the calibration plots for all the models and all causes of death. The calibration plots show very similar performance of the models for all causes of death.

### Decision curve analysis

**Fig. 3:**
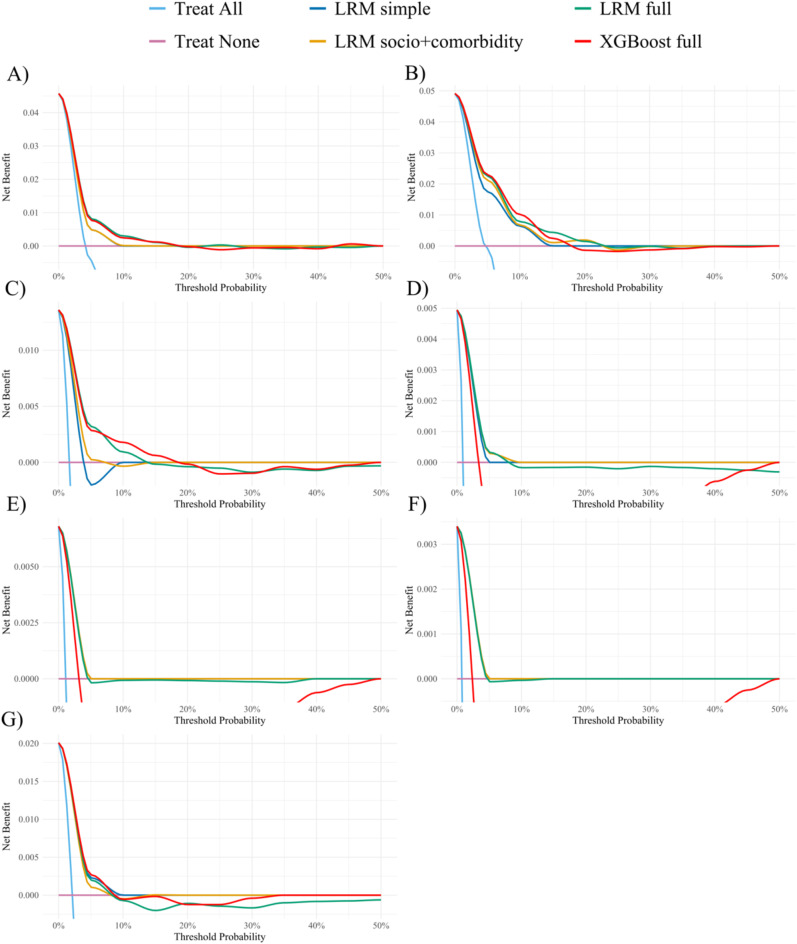
Decision curve for validation of the prediction models developed to estimate the risk of dying from specific causes based on the logistic regression models (LRM) and extreme gradient boosting (XGBoost) models for dying from (**A**) cancer, (**B**) cardiovascular diseases, (**C**) dementia or Parkinson’s disease, (**D**) diabetes, (**E**) digestive diseases, (**F**) psychiatric diseases or suicide, and (**G**) respiratory diseases. The x-axis shows the probabilities which when exceeded, individuals are classified as high risk of dying from a specific disease within 3 years of the index date. The y-axis shows the proportion of accurately diagnosed and treated individuals after subtracting the weighted false positives for each threshold probability. For example a net benefit of 0.01 means that at this threshold probability, we find one true positive out of 100 individuals without unnecessarily intervening on those.

Figure 3 shows the decision curves for the developed models for dying from cancer, CVD, dementia or Parkinson’s disease and respiratory diseases. The figure illustrates different scenarios based on the developed models and two additional scenarios *treat all* and *treat none*. Treat all describes a scenario, where all individuals will experience treatment without adhering to a prediction model and the treat none scenario describes a scenario where all individuals would not experience treatment without adhering to a prediction model. Overall, the figure shows that including the socioeconomic factors and comorbidity as predictors does not improve the net benefit. Including the different healthcare expenditures as predictors improves the new benefit for dying from cancer and dementia or Parkinson’s disease. The difference is not as clear for dying from the other causes of death. The XGBoost models does not show higher net benefit apart from dying from dementia or Parkinson’s disease between the threshold probabilities around 10–20%. The decision curve analysis further showed that the full models have close to zero net benefit from 10–20% threshold probability and above. Furthermore, it also shows that for dying from diabetes, digestive diseases and psychiatric diseases or suicide the net benefit is zero already from threshold probabilities at around 5%.

## Discussion

### Main findings

In this extensive cohort study encompassing married older adults, we explored the link between spousal bereavement and cause-specific mortality within a causal framework. We found that older males who had experienced bereavement had higher risks of dying from cardiovascular disease (CVD), digestive diseases, psychiatric disorders or suicide, and respiratory diseases compared to those who did not experience bereavement. Correspondingly, looking at the absolute risk differences for females, we found that experiencing bereavement was associated a higher risk of dying from CVD, psychiatric diseases or suicide and respiratory diseases. The results showed higher absolute risks among bereaved and non-bereaved older males compared to females, but the absolute risks were small for both sexes. This is the first study to our knowledge, that presents absolute risks of dying from a specific cause following spousal bereavement, highlighting the small risk differences for older adults who have not experienced bereavement to those who have.

This study evaluated whether healthcare expenditure data could improve the prediction of cause-specific mortality. While our models showed reasonable discrimination for specific causes of death, such as diabetes, respiratory diseases, and dementia/Parkinson’s disease, the addition of healthcare expenditures and other predictors did not improve prediction accuracy compared to simpler models. This suggests that despite having access to comprehensive healthcare utilization data, accurately predicting individual cause-specific mortality remains challenging.

### Comparison to previous literature

All previous studies investigating the link between spousal bereavement and mortality have investigated the link between spousal bereavement and cause-specific mortality with regression analysis estimating the magnitude of the association on a relative scale^13,15–17^. In the present study we have deployed a counterfactual approach using G-estimation in a matched cohort, which supports the hypothesis of causal association. Overall, our findings are in line with the results from previous studies on the association between spousal bereavement and cause-specific mortality among older adults^13,15–17^ indicating consistency. Generally the studies adjusted for measurements of sociodemographic and different measures for health status, however one study only adjusted for age and sex^17^ and one study lacked information on health status^16^. Two Norwegian studies^16,17^ found higher risks of dying from circulatory diseases, respiratory diseases, and external causes for males and females who had experienced bereavement compared to those who had not, but not from cancer. This indicates that the findings of this study also could be applicable to other Scandinavian welfare states. The results for dying from Dementia or Parkinson’s disease are in line with previous findings from Elwert and Christakis^13^. Blanner and colleagues did not find an association for neurological diseases and mental disorders among males, which could be explained by the differences in the grouping of diseases as we found a positive association for dying from psychiatric diseases or suicide, but a negative association for dying from Dementia or Parkinson’s disease. However, a hypothesized explanation for this finding could be underdiagnosis of Dementia among the bereaved older adults. For dying from cancer, all studies^13,15–17^ found an association, whereas we did not. There are several differences in the study design from the previous studies and ours. We have taken a causal framework approach to the analysis, used matching, and adjusted for several confounders that previous studies have not.

Two previous studies^10,18^, based on the same source population of older adults who have experienced bereavement as the present study, found that time series of healthcare expenditures were a strong predictor of all-cause mortality. They found evidence that the indicators capture the dynamics of human resilience because they found indication of increased frailty with age. The use of healthcare expenditures was modelled differently in our analysis as we included each type of healthcare expenditure as a separate predictor of an average expenditure one year prior to the index date. The authors of the previous studies used time series of healthcare expenditures and several different measurements of the trends of the expenditures^18^ and aggregated time series of healthcare expenditures^10^. They found that the healthcare expenditures measured closest to the index date were the most predictive of all-cause mortality. Therefore, limiting the healthcare expenditures to a one-year average in our analysis is a minor possible limitation, but the choice has practical benefits, as the model needs less compute. While the previous studies showed that healthcare expenditures were strong predictors of all-cause mortality their limited performance in our study suggests information on healthcare expenditures may serve better as a general indicator of overall health status rather than capturing the specific patterns needed to predict specific causes of death.

### Strengths and limitations

The present study has several strengths. One of the major strengths is the sample size of the study, in which we leveraged nationwide register data with high validity^20,30,31^.

Another strength is the study design. We chose to exclude unmarried individuals for the probability of everyone experiencing bereavement to be non-zero. This was done with to ensure positivity, which is necessary to draw causal conclusions. The findings are limited to married older adults and are, therefore, not applicable to unmarried cohabitating couples. We assessed the older adults based on the bereavement group they were initially assigned to. Thus, we did not take remarriage into account or that the non-bereaved could experience bereavement during the follow-up. However, remarriage at this age is not common and it would be unlikely to significantly change the direction of the effect.

We used matching of bereaved and non-bereaved as it allowed us to use the average expenditure one year prior to index date for all individuals and to have common follow-ups for all. Furthermore, we used matching to comply with the assumption of exchangeability. The matching has potential to limit the risk of unmeasured confounding, as we hope that the older adults are similar in more ways than in those matched upon. However, there is a risk of not capturing some of the differences between the bereaved and non-bereaved which could bias the results. Furthermore, married couples share endless risk factors such as smoking, diet, sleeping habits that during a lifetime could affect both the dead spouse, but also the bereaved. Some of these risk factors might be reflected in the healthcare expenditures. With respect to the ATE estimation, different ATEs could be obtained under other distributions of the covariates X, which could introduce another source of bias.

The present study has several methodological strengths beyond the matched study population. Using the average treatment effect analysis for estimating the average bereavement effect allows for a natural interpretation of potential outcomes supporting the hypothesis of causal associations. The counterfactual approach reduces the risk of confounding as the effect of bereavement is investigated in the entire study population as what would have been the potential outcome, had all older adults experienced bereavement and what would have happened if they had not experienced bereavement.

No previous study investigating spousal bereavement in older adults has to our knowledge presented their findings as absolute risk estimates. This is a great strength of this study as ratio estimates can be misleading, especially if the risks are small. Absolute risks can be helpful for decision making and for prevention purposes as the potential number of people to help in an intervention is clear.

The healthcare expenditures were used as a proxy for the health of the older adults. In Denmark, where the healthcare system is tax based and out-of-pocket payments only accounts for around 15% of health care expenditures^32^ (see supplementary text: *Setting*), one could argue that there is free and equal access to the healthcare system and, thus, we would have captured the heath of all individuals. However, studies from Denmark have shown that individuals with lower socioeconomic status^33^ or from immigrant background^34^ are less likely to utilize the healthcare system when ill. It is a strength of the present study to include these factors (affluence, immigration status) in the prediction model, as we might be able to capture this complex dynamic.

The present study uses individual person-level data from a cohort of older adults, which is the ideal type of data for developing a prediction model^26^. We used several metrics to evaluate model performance, whereas several previous studies have been criticised for only evaluating the performance based on the AUC^35^. In the present study, we assessed both the model’s discriminative ability, calibration and presented decision curve analysis. This is a strength of this study, as poor calibration of a model can make the predictions misleading and thereby reduce the utility of the model^36^.

Prediction models can have practical applications in either the healthcare system, such as at the general practitioner, or municipality for resource allocation. By identifying individuals at heightened risk of dying for specific causes of death, the model could serve as a tool for prioritizing resources, enabling targeted interventions. However, given our prediction models’ limited predictive accuracy, caution about their potential clinical applications is required. The modest performance of even our most complex models suggests that using these predictions for resource allocation or intervention targeting would be premature. Future research should focus on developing more accurate prediction methods before considering clinical implementation.

In conclusion, the findings of our study indicated a higher risk, although still small of magnitude, of death from CVD, digestive diseases, psychiatric diseases or suicide, and respiratory diseases among older males and death from CVD, psychiatric diseases or suicide and respiratory diseases among older females living in Denmark who experienced spousal bereavement, compared to those had not. However, for dying from dementia or Parkinson’s disease, the findings showed a lower number of deaths associated with bereavement. The developed prediction models showed low predictive performance for cause-specific mortality even when incorporating comprehensive information on healthcare expenditures and sociodemographic data.

## Electronic supplementary material

Below is the link to the electronic supplementary material.

Supplementary Material 1

## Data Availability

The data for the analysis is individual level data from Statistics Denmark. The data can only be shared under specific conditions. According to Danish law, scientific organizations can be authorized to work with data within Statistics Denmark and can provide access to individual scientists inside and outside of Denmark. Data are available via the Research Service Department at Statistics Denmark: www.dst.dk/da/TilSalg/Forskningsservice for researchers who meet the criteria for access to confidential data.
