# Supplementary material for: Cause-specific mortality after spousal bereavement in a Danish register-based cohort
Source: Sci Rep. 2025 Feb 20;15:6240. doi: 10.1038/s41598-025-90657-1 (PMC11842573; doi:10.1038/s41598-025-90657-1)
Supplement: Supplementary file 1 — Supplementary Material 1 [file 41598_2025_90657_MOESM1_ESM.docx]

Supplementary

**Paper**

*“Cause-specific mortality after spousal bereavement in a Danish register-based cohort”* published in Scientific Reports.

**Authors**

Mathilde Marie Brünnich Sloth^1,2*^, Johannes Hruza^1^, Laust Hvas Mortensen^1,2,4^, Samir Bhatt^1,3+^, and Alexandros Katsiferis^1,2+^

**Affiliations**

^1^ Section of Epidemiology, Department of Public Health, University of Copenhagen

^2^ Data Science Lab, Statistics Denmark, Copenhagen, Denmark

^3^ Department of Infectious Disease Epidemiology, Imperial College London, London, United Kingdom

^4^ The ROCKWOOL foundation, Ny Kongensgade 6, 1472 Copenhagen K, Copenhagen, Denmark

+ Joint Senior Authors

***** E-mail: mathilde.sloth@sund.ku.dk

**Supplementary Text 1: Setting**

The study included individuals living in Denmark. Denmark is a Northern European welfare state with 5,883,562 inhabitants [1]. In Denmark, general practitioners (GPs) play a crucial role as intermediaries, directing patients to hospitals, and specialist treatment [2]. While a significant portion of healthcare services is provided at no cost, certain healthcare services, such as psychological therapy without a GP referral, may require out-of-pocket payments. Additionally, prescription medications are typically partially subsidized [3]. Home care services, which encompass practical help and personal care, are provided by the municipality and are generally free of charge for elderly individuals. Home nursing is also offered at no cost when prescribed by a GP. However, when elderly individuals require extensive care and move into a care home, they are responsible for individual expenses such as residential facilities and food. Nursing and healthcare services in care homes are provided free of charge [4].

[1] Statistics Denmark. Befolkningstal, https://www.dst.dk/da/Statistik/emner/borgere/befolkning/befolkningstal (2022, accessed 2 June 2022).

[2] Ministry of Health. *Healthcare in Denmark: an overview*. 1.2. Copenhagen, Denmark: Ministry of Health, 2017.

[3] Danish Medical Agency. Reimbursement and prices. *Danish Medicines Agency*, https://laegemiddelstyrelsen.dk/en/reimbursement/ (2019, accessed 2 June 2022).

[4] Healthcare Denmark. Elderly Care. *Healthcare Denmark*, https://healthcaredenmark.dk/national-strongholds/elderly-care/ (n.d., accessed 31 October 2023).


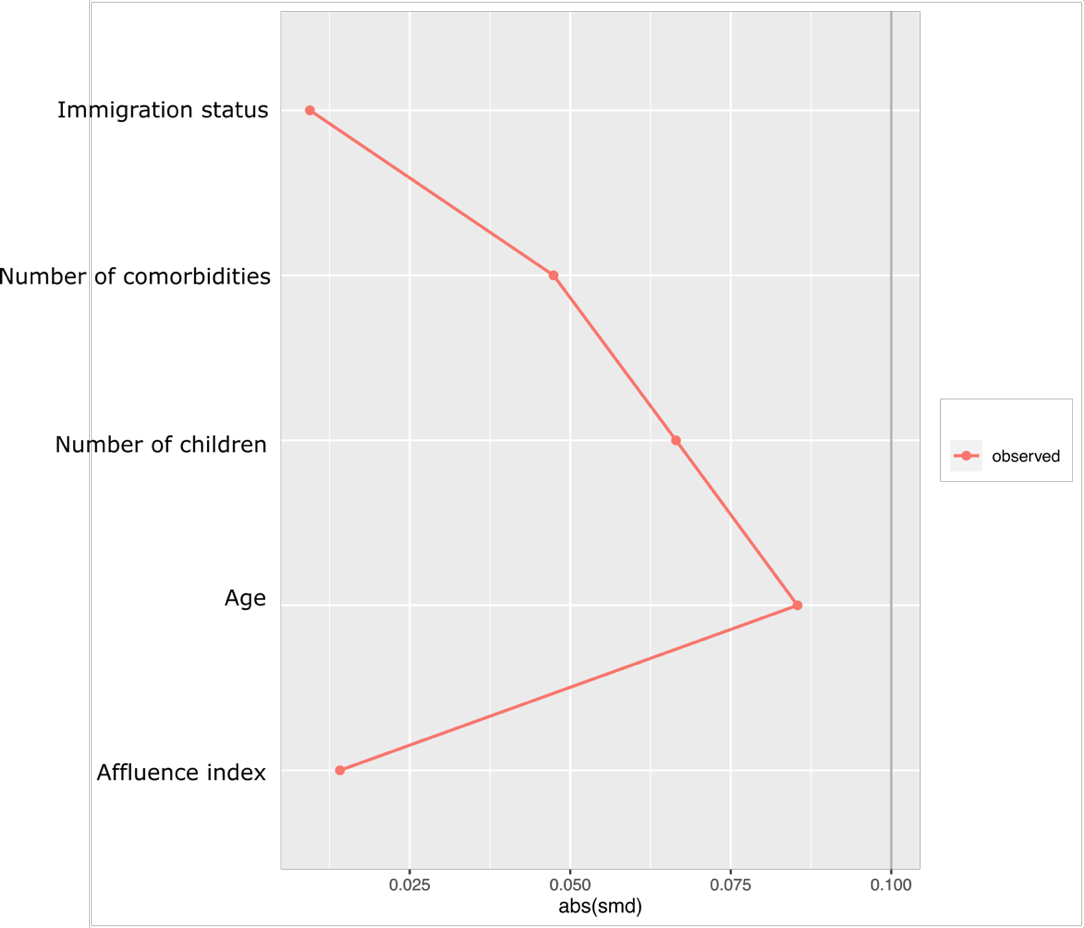


**Supplementary Figure 1 (a): Standardised mean difference of the propensity score matched study population for males**

**
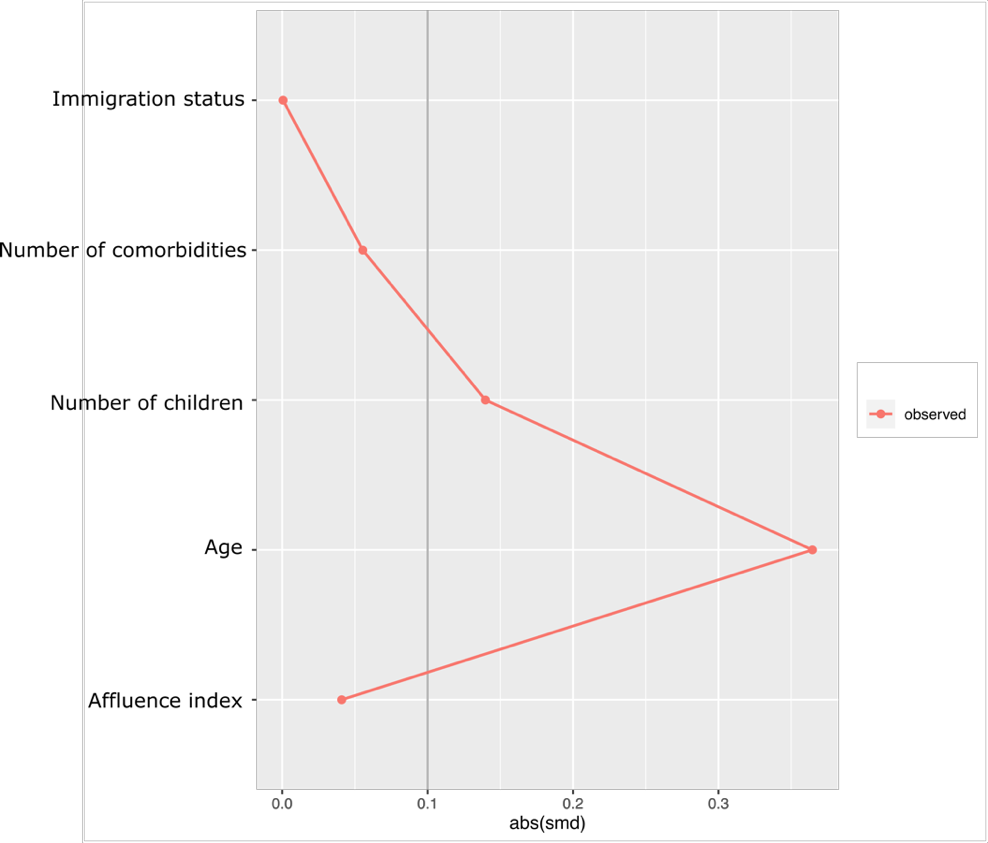
**

**Supplementary Figure 1 (b): Standardised mean difference of the propensity score matched study population for females**

**Supplementary Table 1: Categorization of causes of death [5]**

| **Causes of death** | **ICD-10** |
| --- | --- |
| **Cancer** | All C diagnosis |
| **Diabetes** | E10-E14 |
| **Dementia and Parkinson’s disease** | Dementia: F00, G30, F01, F02.0, F03.9, G31.8b, G31.8E, G31.9, G31.0b  Parkinson: G20, G21, G22, F02.3 |
| **Cardiovascular diseases** | All I diagnosis |
| **Diseases related to the digestive system** | All K diagnosis |
| **Psychiatric diseases and suicide** | X60-X84  All F diagnosis, except dementia (F00, F01, F02.0, F03.9) |
| **Respiratory diseases** | All J diagnosis |

[5] World Health Organization. International Statistical Classification of Diseases and Related Health Problems 10th Revision. ICD-10 Version:2019, https://icd.who.int/browse10/2019/en (2019, accessed 28 November 2023).

**
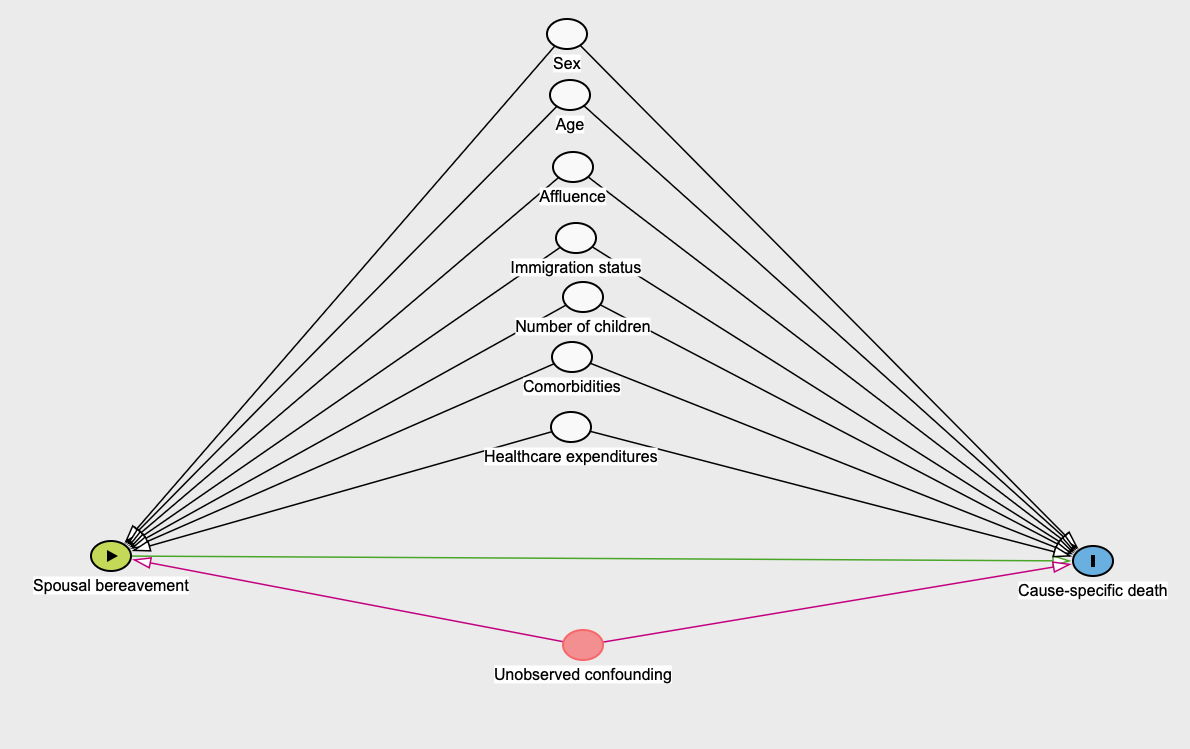
**

**Supplementary Figure 2: Directed Acyclic Graph of the association investigated between spousal bereavement and cause-specific mortality**

**Supplementary Table 2. The most common causes of death within each category**

| **Cause of death** |  | **Number of deaths, n(%)** |
| --- | --- | --- |
| **Cancers** | Other | 2749 (21.9) |
|  | Lung | 2720 (21.6) |
|  | Digestive organs | 2304 (18.3) |
|  | Male genitals | 1429 (11.4) |
|  | Breast and female genitals | 1414 (11.2) |
|  | Colon | 1223 (9.7) |
|  | Urinary tract | 733 (5.8) |
| **Cardiovascular**  **diseases** | Ischaemic heart disease | 3348 (30.3) |
|  | CVA and stroke | 3005 (27.2) |
|  | Other | 1535 (13.9) |
|  | Heart failure | 862 (7.8) |
|  | Diseases of arteries, arterioles, and capillaries | 846 (7.7) |
|  | Atrial fibrillation and flutter | 735 (6.6) |
|  | Hypertensive disease | 727 (6.6) |
| **Dementia or**  **Parkinson’s disease** | Unspecified dementia | 3895 (49.2) |
|  | Alzheimer | 2364 (29.9) |
|  | Parkinson’s | 947 (12.0) |
|  | Vascular dementia | 703 (8.9) |
| **Diabetes** | Diabetes | 609 (100) |
| **Digestive diseases** | Other | 353 (26.7) |
|  | Diseases of oesophagus, stomach, and duodenum | 259 (19.6) |
|  | Diseases of liver | 211 (15.9) |
|  | Diseases of gallbladder, biliary tract, and pancreas | 197 (14.9) |
|  | Paralytic ileus and intestinal obstruction without hernia | 154 (11.6) |
|  | Vascular disorder of intestine | 79 (6.0) |
|  | Diverticular disease of intestine | 71 (5.4) |
| **Psychiatric diseases or suicide** | Suicide | 221 (56.1) |
|  | Mental and behavioural disorder due to use of alcohol | 96 (24.4) |
|  | Other | 42 (10.7) |
|  | Uni or bipolar depression | 35 (8.9) |
| **Respiratory**  **diseases** | Chronic obstructive pulmonary disease | 2934 (55.3) |
|  | Influenza and pneumonia | 1640 (30.9) |
|  | Other respiratory diseases | 735 (13.8) |

**Supplementary Table 3: Performance of the prediction models for cause-specific mortality in AUC and Brier Scores with 95% confidence intervals**

| **Cause of death** | **Model** | **AUC [CI] (%)** | **Brier Score [CI] (%)** | **IPA** |
| --- | --- | --- | --- | --- |
| Cancer | LRM null | 50.00 [50.00;50.00] | 4.37 [3.71;5.04] | 0.00 |
|  | LRM simple | 62.26 [57.71;66.81] | 4.34 [3.68;5.00] | 0.01 |
|  | LRM socio + comorbidity | 64.64 [60.33;68.96] | 4.32 [3.67;4.98] | 0.01 |
|  | LRM full | 66.37 [61.78;70.95] | 4.29 [3.65;4.93] | 0.02 |
| Cardiovascular diseases | LRM null | 50.00 [50.00;50.00] | 4.67 [4.01;5.34] | 0.00 |
|  | LRM simple | 73.99 [70.24;77.74] | 4.50 [3.88;5.12] | 0.04 |
|  | LRM socio + comorbidity | 76.38 [72.68;80.07] | 4.45 [3.84;5.05] | 0.05 |
|  | LRM full | 78.60 [75.04;82.16] | 4.38 [3.79;4.98] | 0.06 |
| Dementia or Parkinson’s disease | LRM null | 50.00 [50.00;50.00] | 1.34 [0.96;1.73] | 0.00 |
|  | LRM simple | 72.96 [65.84;80.09] | 1.33 [0.96;1.71] | 0.01 |
|  | LRM socio + comorbidity | 74.04 [67.13;80.95] | 1.33 [0.96;1.70] | 0.01 |
|  | LRM full | 87.08 [82.35;91.80] | 1.33 [0.98;1.68] | 0.01 |
| Diabetes | LRM null | 50.00 [50.00;50.00] | 0.49 [0.25;0.73] | 0.00 |
|  | LRM simple | 74.14 [61.14;87.13] | 0.49 [0.25;0.73] | 0.00 |
|  | LRM socio + comorbidity | 83.69 [77.37;90.01] | 0.49 [0.25;0.72] | 0.01 |
|  | LRM full | 88.62 [83.31;93.92] | 0.51 [0.27;0.75] | -0.03 |
| Digestive diseases | LRM null | 50.00 [50.00;50.00] | 0.68 [0.40;0.95] | 0.00 |
|  | LRM simple | 64.36 [52.98;75.74] | 0.67 [0.40;0.95] | 0.00 |
|  | LRM socio + comorbidity | 66.98 [54.91;79.04] | 0.67 [0.40;0.95] | 0.00 |
|  | LRM full | 67.36 [55.57;79.16] | 0.68 [0.40;0.96] | -0.01 |
| Psychiatric diseases or suicide | LRM null | 50.00 [50.00;50.00] | 0.34 [0.14;0.54] | 0.00 |
|  | LRM simple | 71.37 [55.13;87.61] | 0.34 [0.14;0.54] | 0.00 |
|  | LRM socio + comorbidity | 61.32 [44.24;78.40] | 0.34 [0.14;0.54] | 0.00 |
|  | LRM full | 66.97 [46.98;86.95] | 0.34 [0.14;0.54] | 0.00 |
| Respiratory diseases | LRM null | 50.00 [50.00;50.00] | 1.97 [1.51;2.43] | 0.00 |
|  | LRM simple | 77.88 [73.14;82.61] | 1.93 [1.48;2.37] | 0.02 |
|  | LRM socio + comorbidity | 77.34 [72.99;81.69] | 1.94 [1.50;2.38] | 0.01 |
|  | LRM full | 81.86 [78.03;85.68] | 2.01 [1.58;2.45] | -0.02 |

**Supplementary Table 4: Model comparison of logistic regression models using delta AUC and delta Brier Score with 95% confidence intervals**

| Cause of death | Model | Reference | Delta AUC [95% CI] | Delta Brier Score [95% CI] |
| --- | --- | --- | --- | --- |
| Cancer | LRM Simple | LRM Null | 12.26 [7.71;16.81] | -0.03 [-0.05;-0.01] |
|  | LRM Socio + comorbidities | LRM Simple | 2.38 [-0.9;5.67] | -0.02 [-0.04;0.00] |
|  | LRM Full | LRM Socio+ comorbidities | 1.72 [-1.86;5.31] | -0.03 [-0.12;0.07] |
| Cardiovascular diseases | LRM Simple | LRM Null | 23.99 [20.24;27.74] | -0.18 [-0.28;-0.08] |
|  | LRM Socio + comorbidities | LRM Simple | 2.38 [0.26;4.50] | -0.05 [-0.12;0.01] |
|  | LRM Full | LRM Socio+ comorbidities | 2.22 [0.65;3.80] | -0.06 [-0.14;0.01] |
| Dementia or Parkinson’s disease | LRM Simple | LRM Null | 22.96 [15.84;30.09] | -0.01 [-0.03;0.01] |
|  | LRM Socio + comorbidities | LRM Simple | 1.08 [-2.00;4.15] | 0.00 [-0.01;0.01] |
|  | LRM Full | LRM Socio+ comorbidities | 13.04 [6.83;19.24] | 0.00 [-0.12;0.11] |
| Diabetes | LRM Simple | LRM Null | 24.14 [11.14;37.13] | 0.00 [0.00;0.00] |
|  | LRM Socio + comorbidities | LRM Simple | 9.55 [-4.06;23.17] | 0.00 [-0.01;0.00] |
|  | LRM Full | LRM Socio+ comorbidities | 4.93 [-1.91;11.76] | 0.02 [-0.02;0.06] |
| Digestive diseases | LRM Simple | LRM Null | 14.36 [2.98;25.74] | 0.00 [0.00;0.00] |
|  | LRM Socio + comorbidities | LRM Simple | 2.61 [-7.87;13.10] | 0.00 [0.00;0.00] |
|  | LRM Full | LRM Socio+ comorbidities | 0.39 [-6.72;7.50] | 0.01 [0.00;0.01] |
| Psychiatric diseases or suicide | LRM Simple | LRM Null | 21.37 [5.13;37.61] | 0.00 [0.00;0.00] |
|  | LRM Socio + comorbidities | LRM Simple | -10.05 [-20.74;0.64] | 0.00 [0.00;0.00] |
|  | LRM Full | LRM Socio+ comorbidities | 5.65 [-11.08;22.38] | 0.00 [0.00;0.00] |
| Respiratory diseases | LRM Simple | LRM Null | 27.88 [23.14;32.61] | -0.04 [-0.07;-0.02] |
|  | LRM Socio + comorbidities | LRM Simple | -0.53 [-3.77;2.70] | 0.01 [-0.01;0.04] |
|  | LRM Full | LRM Socio+ comorbidities | 4.51 [-0.16;9.19] | 0.07 [-0.01;0.16] |

**Supplementary Table 5: Model comparison of the logistic regression model (LRM full) to extreme gradient boosting (XGBoost full) presented with delta AUC and delta Brier score with 95% confidence intervals**

| Cause of death | model | reference | Delta AUC [95% CI] | Delta Brier Score [95% CI] |
| --- | --- | --- | --- | --- |
| Cancer | XGBoost Full | LRM Full | -1.12 [-4.27;2.04] | 0.01 [-0.07;0.09] |
| Cardiovascular diseases | XGBoost Full | LRM Full | 0.81 [-1.11;2.73] | 0.05 [-0.02;0.11] |
| Dementia and Parkinson’s disease | XGBoost Full | LRM Full | 0.05 [-3.02;3.13] | -0.04 [-0.13;0.04] |
| Diabetes | XGBoost Full | LRM Full | -2.49 [-9.16;4.17] | -0.02 [-0.06;0.02] |
| Digestive diseases | XGBoost Full | LRM Full | -4.16 [-11.93;3.62] | -0.01 [-0.01;0.00] |
| Psychiatric diseases or suicide | XGBoost Full | LRM Full | -14.91 [-32.16;2.34] | 0.00 [0.00;0.00] |
| Respiratory diseases | XGBoost Full | LRM Full | 0.76 [-2.21;3.72] | -0.07 [-0.15;0.00] |

**
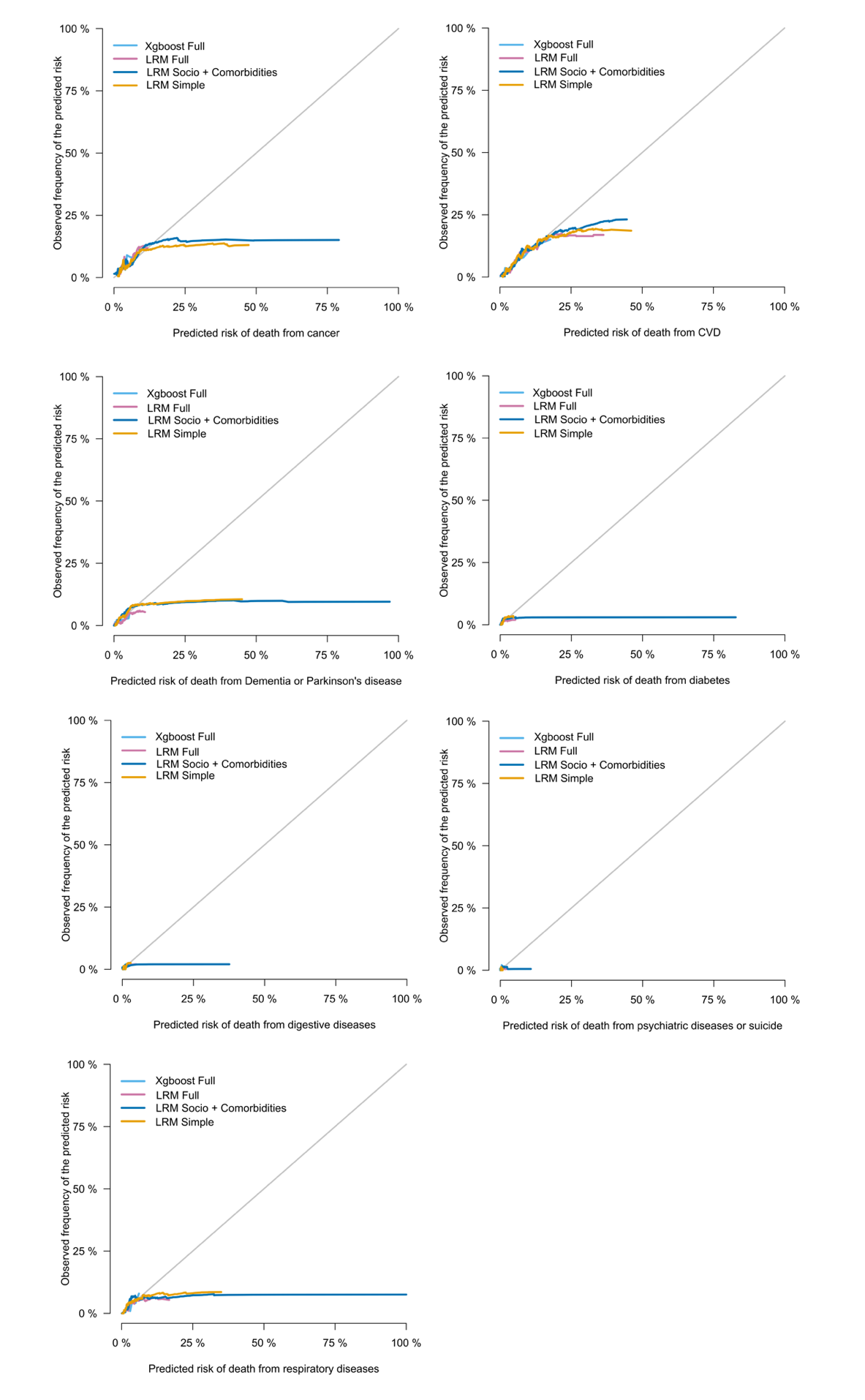
**

**Supplementary Figure 3: Calibration plots of the logistic regression and XGBoost models**
